# Breaking through lag: enhancing intradermal electro-osmotic flow and the future of delay-free continuous glucose monitoring

**DOI:** 10.3389/fbioe.2025.1717650

**Published:** 2026-01-16

**Authors:** Davide Ciarrocchi, Ruben Van den Eeckhoudt, Nurul Izni Rusli, Alessandro Zompanti, Simone Grasso, Lazzaro di Biase, Marco Santonico, Giorgio Pennazza, Irene Taurino

**Affiliations:** 1 Department of Science and Technology for Sustainable Development and One Health, Unit of Electronics for Sensor Systems, University Campus Bio-Medico di Roma, Rome, Italy; 2 KU Leuven, Micro- and Nanosystems (MNS), Department of Electrical Engineering (ESAT), Leuven, Belgium; 3 Department of Mechatronics, Faculty of Electrical Engineering & Technology, Universiti Malaysia Perlis, Perlis, Malaysia; 4 Department of Engineering, Unit of Electronics for Sensor Systems, University Campus Bio-Medico di Roma, Rome, Italy; 5 Research Unit of Neurology, Neurophysiology and Neurobiology, Department of Medicine and Surgery, Università Campus Bio-Medico di Roma, Rome, Italy; 6 Brain Innovations Lab, Università Campus Bio-Medico di Roma, Rome, Italy; 7 KU Leuven, Department of Physics and Astronomy (HF), Leuven, Belgium; 8 KU Leuven, Department of Electrical Engineering (ESAT-MNS), Leuven, Belgium

**Keywords:** continuous glucose monitoring, diabetes, electrical stimulation, electro-osmotic flow, interstitial fluid, lag time reduction, low current density, microfluidic chip

## Abstract

Diabetes mellitus represents one of the most widespread chronic diseases globally, characterized by alterations in glucose metabolism that require constant monitoring of blood glucose levels. Traditionally, blood testing has been the standard for glucose monitoring; however, interstitial fluid has emerged as a viable alternative, due to its less invasive nature which enhances user comfort. Despite improvements in technology, the accuracy of currently available continuous glucose monitors remains a concern, particularly when the rate of change is higher, such as in hypoglycemic and hyperglycemic ranges. Effective management of hypoglycemia relies on the monitor’s ability to provide precise and specific readings when blood glucose levels drop dangerously low. In this context, the demand for heightened accuracy is paramount to timely alert users to impending hypoglycemic events. The inaccuracies of these sensors are attributed to the dynamics of the sample analysis. Specifically the interstitial fluid experiences a delay in concentration due to the diffusion process from capillary blood to interstitial fluid. In this study, we developed a microfluidic device that simulates the diffusion dynamics from capillary glucose to interstitial fluid. We demonstrate the reduction of lag time diffusion from 20 min to 5 min by increasing dermal electro-osmotic flow, which generates convection that transports glucose faster than diffusion, thus resulting in lower lag times. These findings highlight the potential of inciting electro-osmotic flow for improving the responsiveness and accuracy of CGMs, ultimately enhancing diabetes management for users.

## 1 Introduction

Type 1 diabetes is a metabolic disorder characterized by elevated blood glucose levels, which can lead to serious complications such as heart disease, kidney disease, retinopathy, and neuropathy (Harding et al., 2019; Galicia-Garcia et al., 2020). The global prevalence of diabetes has risen dramatically, with over 415 million people affected worldwide. This alarming increase highlights the urgent need for effective management strategies and innovative treatment solutions (Ogurtsova et al., 2017).

The traditional capillary blood glucose meters measure the concentration of glucose from capillary blood samples obtained by the patient. This procedure, in addition to being painful and inconvenient, results in sampling a limited number of glucose values throughout the day, leaving the patient without proper monitoring during the day and especially at night, which can be quite critical in some cases.

Wearable devices for continuous self-monitoring of glucose can play a crucial role in the management of the disease. While traditional glucose meters use capillary blood, most commercial Continous Glucose Monitoring (CGM) devices measure glucose from interstitial fluid (ISF) localized in the subcutaneous adipose tissue including Freestyle Libre, Dexcom G7 (subcutaneous needle) and Eversense from Senseonics (subcutaneous sensor), which can be accessed using a minimally invasive approach. Many compounds, such as glucose, are transported from the blood into the cells via ISF (Scallan and Huxley, 2009; Sansalone et al., 2013). The glucose concentration in ISF strongly correlates with the blood glucose concentration, but the sampling of ISF non-invasively is challenging due to the barrier function of the skin. Thus, all approaches based on ISF that have reached commercial success are based on needles that penetrate the skin and reach the ISF in the dermis (Cengiz and Tamborlane, 2009; Dye et al., 2010). For instance, persons with type-1 diabetes who use CGM devices are less prone to hypoglycemia (low blood sugar) and hyperglycemia (high blood sugar) (Fokkert et al., 2019; Charleer et al., 2020), stay longer within the target glucose range (Šoupal et al., 2020; Šoupal et al., 2016), and have better glycated hemoglobin (HbA1c) levels.

Other approaches for non-invasive CGM under active research include sampling of the interstitial fluid with reverse iontophoresis (Rao et al., 1993; Pu et al., 2021; Leboulanger et al., 2004; Ching and Connolly, 2008; Chin et al., 2008; Wang et al., 2025), magnetic fields (Hakala et al., 2022), ultrasound (Mitragotri Samir et al., 2000; Mitragotri Sami et al., 2000), and detection of glucose through optical methods (Diessel et al., 2005; Hsieh and Zahn, 2005), radio waves (Hanna et al., 2020) and non-enzymatic glucose sensors (Yu et al., 2024; Benedetti et al., 2024; Dissanayake et al., 2025). Recent advancements in technology have removed the need for calibrating CGMs with fingerprick glucose measurements. However, the accuracy of all commercially available CGMs remains lowest in the hypoglycemic range, where the demand for accuracy is critical for effectively functioning as an alarm for hypoglycemia. Both invasive and non-invasive methods for CGM face the common challenge of lag time between the concentration of glucose in ISF and in blood. The glucose concentration in the ISF, which depends on concentration differences that change over time due to physical activities and insulin doses (Scuffi et al., 2012; Davey et al., 2010), reaches about 70% of the glucose level in the blood. According to the literature (Davey et al., 2010), there is a delay of approximately 15–20 min in this process and is influenced by factors such as local blood flow, tissue perfusion and ISF permeability (Schmelzeisen-Redeker et al., 2015). The mismatch between glucose readings from CGMs and glucose concentration in blood is of concern, particularly in the hypoglicemic range, where CGMs time lag causes CGMs glucose readings to deviate from actual glucose levels by more than 2.2 mM (40 mg/dL) in response to rapid rates of decline in glucose concentration (Davey et al., 2010). In the case of CGM sensors, the delay depends on various contributions, including physiological and technological factors. The physiological time delay in glucose sensing primarily arises from the time it takes for glucose to diffuse through capillary walls and the interstitial space before reaching the sensor (Schmelzeisen-Redeker et al., 2015). Notably, there is significant variability in these time delays among individuals and across different CGM systems. In addition to physiological delays, technological time delays can occur and are due to calculations before the results are displayed. Another contribution of delay is related to the filtering techniques used to mitigate data noise, with reported technological delays ranging from 3 to 12 min (Keenan et al., 2009; Rebrin et al., 2010). These delays are also attributed to glucose diffusion through protective membranes and the sensor’s reaction speed, such as enzymatic activity in the case of electrochemical glucose sensors currently used in CGM systems, which generally accounts for an additional few minutes. Reports indicate a wide range of overall time delays, from 5 to 40 min (Keenan et al., 2009; Rebrin et al., 2010), which may stem from differences in CGM systems or experimental conditions. However, much of the literature fails to address interindividual and intraindividual differences in these delays due to insufficient data. Additionally, the effects of glycemic ranges and patient-specific factors—such as physical activity—on time delays remain largely unexplored (Schmelzeisen-Redeker et al., 2015). This lag can significantly affect the timely detection of hypoglycemia and hyperglycemia, which are critical for effective diabetes management (Facchinetti et al., 2013; Dessi et al., 2019; Kovatchev and Cobelli, 2016). Reducing both physiological and technological time delays is essential for enhancing the accuracy and reliability of CGM systems, as these delays contribute considerably to discrepancies between CGM data and actual blood glucose values, ultimately affecting patient outcomes and timely interventions in diabetes management.

Stout et al. (2004) reported a technique for the reduction of physiological lag time with modulated pressure application to enhance local blood flow. However, the current investigation is constrained by methodological limitations, including a restricted dataset and the need for comprehensive empirical validation to assess reproducibility, sustained efficacy, and consistent performance across variable physiological parameters and glycemic trajectories. Electrical stimulation applied externally to the skin has been explored as a strategy to enhance local blood perfusion, thereby indirectly improving glucose equilibration and potentially reducing the lag in CGM measurements (Peters et al., 1998; Indergand and Morgan, 1994). The mechanisms underlying this effect have been attributed to different physiological pathways. Chen et al. (1991) reported that electrical stimulation can induce vasodilation by exciting peripheral nerves, while Tracy et al. (1988) suggested that increased perfusion results from the elevated metabolic demand of electrically induced muscle contractions. These mechanisms act primarily on microcirculatory dynamics, not directly on transdermal molecular transport. In recent years, microneedle (MN)-based platforms have been increasingly combined with reverse iontophoresis to reduce invasiveness and improve sampling efficiency (Kusama et al., 2021; Li et al., 2021; Cheng et al., 2022; Amir et al., 2025). Microneedles create microchannels through the stratum corneum, facilitating transdermal glucose extraction and enhancing electrode–skin coupling. Despite these advances, the time lag in glucose measurement often remains comparable—or even more pronounced—than in commercially available CGM systems, primarily due to diffusion and equilibration limitations within the interstitial space.

**FIGURE 1:**
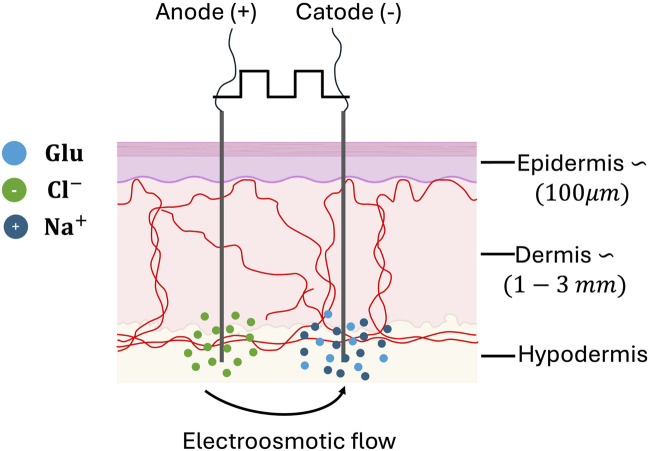
Schematic representation of glucose diffusion enhanced by intradermal electro-osmotic flow.

Considering these premises, this paper presents a microfluidic device that simulate a condition of physiological concentrations difference between the capillary blood and the ISF through a microfluidic channel, a semipermeable membrane, and a reservoir. The device was used to evaluate how the application of electric currents delivered through two electrodes influences the physiological behavior of glucose diffusion between the two compartments. This concept hold potential to enhance the dermal electro-osmotic flow and thus improve the delay between the glucose concentration in capillaries and in the ISF due the physiological diffusion as shown in Figure 1, especially pronounced in case of rapid fluctuations in glucose concentration. This technological concept seamlessly integrates with CGMs, offering potential to enhance patient outcomes.

## 2 Materials and methods

### 2.1 Computational model

The computational model was developed to investigate glucose diffusion variation between the two compartments. The model aimed to characterize the experimental conditions, geometry and parameters governing molecular transport. This model provided a starting point for modeling diffusion between the compartments, effectively translating the real dynamics of diffusion from capillary blood to interstitial fluid within a microfluidic device over a timeframe of 20 min.

The modeling and simulation of the developed device were conducted using COMSOL Multiphysics 6.0 software. The free and porous fluid flow, as well as the transport of diluted species and the laminar flow, was modeled to represent the behavior of the microfluidic setup. The model assumes incompressible flow conditions, with Reynolds numbers ranging from 1.67 to 8.34, confirming deeply laminar flow regime. We developed a 3D model of the geometry consisting of the 200 μm channel ( 1 cm length) and the reservoir, with a diameter of 6 mm . The porous polycarbonate (PC) membrane, positioned between the channel and the reservoir, was modeled with a thickness of 20 μm , as reported in the datasheet. Fluid flow from the channel through the porous membrane into the reservoir was modeled using Darcy’s law, with an hydraulic permeability $k=1.45\times 10^{-14}\;{\text{m}}^{2}$ . The transport of diluted species was described by time-dependent convection-diffusion equations, with a diffusion coefficient of glucose through the membrane obtained through a parametric study based on experimental results and glucose diffusion in water of $6.7\times 10^{-10}\;{\text{m}}^{2}/\text{s}$ . The diffusion coefficient of the membrane was calculated through computational model considering experimental results at timepoint 20 min.

The concentration values within the reservoir were assessed through the volume integral relative to the reservoir, evaluating the glucose concentration in the reservoir at different time points. The laminar flow was set in the microfluidic channel to reproduce the flow conditions defined in the real setup, with the flow established from 20 μL/min to 100 μL/min using a glucose solution at a concentration of 4 mM .

### 2.2 Device fabrication

The fabricated microfluidic chip consists of three distinct layers: (i) the lower microfluidic channel was fabricated in polydimethylsiloxane (PDMS, Sylgard 184, Dow Corning), prepared by mixing the base and curing agent in a 10:1 ratio and cast by soft lithography using an SU-8 mold. The channel had a width of 200 um and was used to simulate the capillary; (ii) the upper microfluidic channel was fabricated identical to the first. A reservoir with a diameter of 6 mm was created along the microfluidic channel by punching a hole through the PDMS using a controlled-diameter punch. This upper channel was used to simulate the ISF; (iii) a commercially available polycarbonate (PC) membrane from Whatman Cyclopore (Maidstone, United Kingdom) with a pore size of 5 um was used as an endothelial mimic layer and located at the interface between the lower microfluidic channel and the reservoir in the upper microfluidic channel. The developed and applied technique involves an optimization of the procedure reported in Jeffrey et al. (2021), including the use of 2% 3-aminopropyl triethoxysilane (APTES) solution, 28% DI water and 70% ethanol.

**FIGURE 2:**
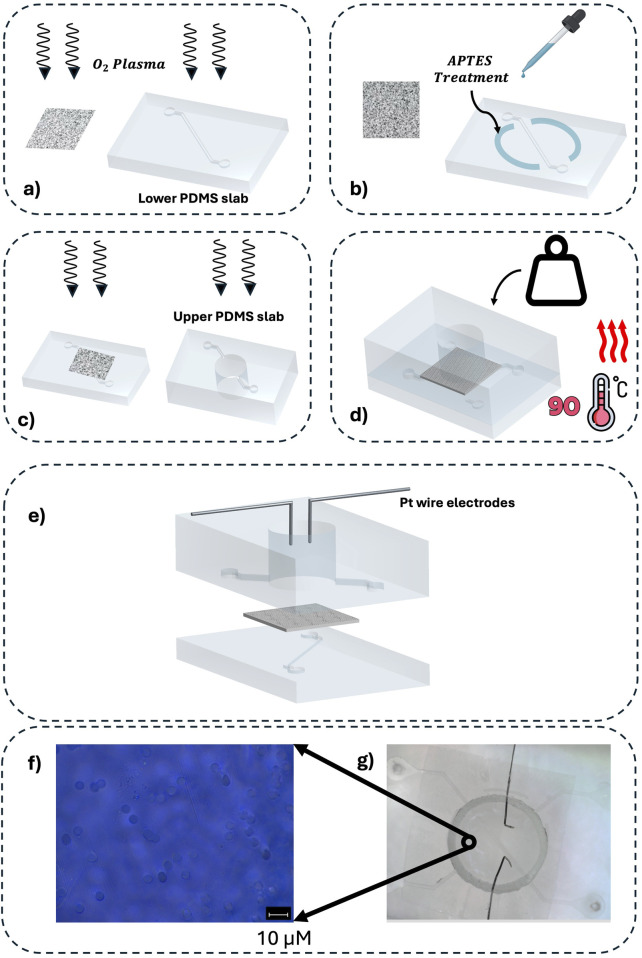
Fabrication workflow of the microfluidic device with the integration of the Pt-wire electrodes. **(a)** Activation of the surface with $O_{2}$ plasma treatment, respectively of the lower PDMS slab and of a face of the PC membrane; **(b)** APTES treatment of the lower PDMS slab in order to cross-link the PC membrane and the microfluidic channel; **(c)** Activation of the surface with $O_{2}$ plasma treatment, respectively of the upper PDMS slab and the lower PDMS slab; **(d)** Post-process thermal treatment at 90 °C for 1 h to strongly bond the sandwich chip; **(e)** 3D schematic illustration of the developed microfluidic chip with the integration of the Pt-wire electrodes; **(f)** Representative SEM images of the PC membrane; **(g)** Top-view image of the microfluidic developed device.

The bonding step was performed using an optimized version of the protocol described in Jeffrey et al. (2021), specifically adapted to avoid any alteration of the membrane’s transport properties. As shown in Figure 2a, the membrane and the lower PDMS slab containing the microfluidic channel were first activated by oxygen plasma (200 mT, 40 sccm of $O_{2}$ , 30 W, 1 min). Subsequently, as illustrated in Figure 2b, a thin layer of the APTES solution (2% APTES, 28% DI water, 70% ethanol) was applied exclusively to the non-exchange perimeter regions of the lower PDMS slab, providing localized surface functionalization to ensure a strong, leak-free bond. Importantly, the membrane surface itself was not exposed to APTES to preserve its native exchange characteristics. As shown in Figure 2c, the membrane was then placed onto the lower PDMS slab with its plasma-treated face contacting the APTES-coated perimeter zones. Finally, both the lower PDMS slab (carrying the microchannel and bonded membrane) and the upper PDMS slab containing the reservoir were plasma-treated and assembled to form the complete three-layer PDMS–membrane–PDMS structure, as depicted in Figure 2d.

Finally, the assembly was chemically bond together by a post bake at 90 °C for 1 h. During the bake a weight of 200 g was applied to ensure a tight contact between the components. This process resulted in a water tight bond ensuring no leakage from the microfluidic channels.

The integration of Pt wire electrodes (diameter = 0.125 mm) was achieved via cuts made on the sides of the reservoir, and the wires were inserted to have approximately 0.9 cm of electrode in contact with the fluid in the reservoir Figure 2e). Once the electrodes were inserted and positioned at a distance of 2 mm, the cuts in the PDMS were sealed with a PDMS mortar.

### 2.3 Electro-diffusion device and experimental setup

Several portable and programmable devices for reverse iontophoresis have been described in the literature, mainly focusing on non-invasive monitoring and transdermal drug delivery (Tak-Shing Ching and Chih, 2014; Tak-Shing Ching et al., 2012; Ching et al., 2005).

The portable electronics used for electro-diffusion is a custom-made apparatus developed by the authors (Zompanti et al., 2024). The system is based on a 32-bit MCU (SAM3X8E), designed for the delivery of dc current, biphasic and monophasic square wave current. The electronic interface incorporates a high-precision voltage-controlled current source coupled with a current mirror and an H-Bridge, providing a wide voltage compliance range of ± 120 V to enable robust and accurate signal control. The current output range is dynamically tunable through a series of selectable resistances that modulate the current gain. The system’s capability to modulate frequency and duty cycle, coupled with microsecond-level timing resolution, allows for precise and flexible electrical stimulation protocols with accuracy in current and voltage parameter manipulation. Additionally, the device is designed to provide continuous monitoring of the injected current and for a safety feature to ground the load when no stimulation is being applied. Unlike voltage-controlled stimulation devices, the precise delivery of current helps to avoid undesirable effects, such as Joule heating and electrolysis, and allows for effective current control over the load. In this specific application, the device was used to deliver controlled current through two platinum electrodes, employing direct current and square waveforms to investigate the effect of the latter on the enhancement of electro-osmotic flow. The applied current of 5 μ A correspond to a current density of 140 μ A/cm^2^, adhering to the applicable safety current standards for patients (0.5 mA/cm^2^) (Kono et al., 2018).

Considering a 0.9 cm length of the electrode in contact with the solution in the reservoir, and applying a current of 5 μ A both continuously and through square wave pulses, with 50% duty cycle, at a frequency of 500 Hz, the resulting current density is:

$$J=\frac{I}{A}=140\;\frac{\mu A}{cm^{2}}$$

where:

-
I=5 μA is the current,
-
d=0.125 mm is the diameter,
-
l=0.9 cm is the Pt wire length,
-
$A=2\pi \left(\frac{d}{2}\right)\left(\frac{d}{2}+l\right)$ is the sum of the lateral surface and the base.

At 500 Hz, the impedance of the medium is relatively low, and with an applied current of 5 μ A, the resulting voltage drop across the load is reduced. Therefore, the compliance voltage can be lowered while still allowing precise current delivery, ensuring safe operation and minimizing the risk of excessive electrode polarization or electrochemical effects.

In the case of current stimulation, the device was connected to the exposed contacts of the microfluidic device and controlled via an interface for current delivery. Concurrently, the voltage values across the load were recorded during the stimulation in order to evaluate the achieved polarization, highlighting the lower impedance encountered at high frequency and furthermore the lower voltage applied delivering the same current. The microfluidic device performance was evaluated by quantifying the transfer of glucose into the reservoir over a range of flow rates ranging from 20 μ L/min to 100 μ L/min and were compared to the predicted results from computational modeling. The inspected flow rates were set using a syringe pump with a 5 mL syringe containing a concentrated solution of 4 mM glucose dissolved in PBS, while the acceptor volume was filled with 80 μ L of PBS. Testing different flow rates allowed us to investigate the glucose concentration in the reservoir after 20 min and to establish an experimental setup that replicates the diffusion of a glucose gradient of 1 mM (approximately 18 mg/dL). Once the optimal flow rate was established through systematic testing, the experiment proceeded to incorporate current stimulation to further evaluate the system’s performance. This methodology allowed for a controlled simulation of physiological glucose variations between the microfluidic channel and the reservoir.

### 2.4 Glucose measurements

Quantitative analysis of glucose concentration was performed using the D-Glucose GOPOD FORMAT enzymatic assay kit from Megazyme Ltd. (Wicklow, Ireland). At predetermined time points, 50 μ L samples were extracted from the reservoir and diluted 1:1 with PBS to achieve the 100 μ L minimum volume required by the assay protocol. Following the manufacturer’s instructions, the samples underwent thermal incubation at 40- 50  °C for 20 min. Absorbance measurements were then conducted at 510 nm using a Shimadzu UV-Vis spectrophotometer to determine the final glucose concentrations.

## 3 Results and discussions

The assembly of the device described in this paper uses a novel way of bonding the PC membranes to the two layers of PDMS. Most commonly, the used techniques include: (i) spin-coating of 1:1 ratio of toluene to PDMS onto glass followed by dipping the PDMS slabs to create a mortar for uniting the interfaces (Chueh et al., 2007); and (ii) functionalization of the membrane through a process based on APTES, (Jeffrey et al., 2021). However, these techniques suffer from several issues: (i) clogging of the channels due to the PDMS mortar; (ii) modification of the membrane properties through APTES treatment, which alters the diffusion characteristics of the PC membrane, leading to a reduction and instability in diffusion.

The bonding method described in the paper did not suffer from channel clogging, as no mortar-like materials were employed during the bonding procedure that could potentially obstruct the microfluidic channels.

Furthermore, membrane properties remained unaltered, since the APTES treatment was selectively applied only to surfaces not involved in fluid exchange between the reservoir and the channel.

Our bonding procedure successfully achieves strong interfacial adhesion between the three layers while crucially preserving the membrane’s diffusion properties - a significant improvement over the previously reported techniques which compromised membrane functionality.

**FIGURE 3:**
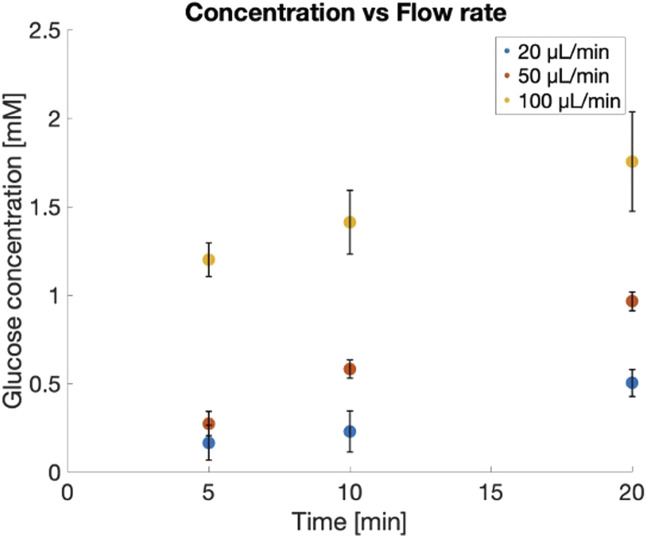
The time-course glucose concentration in the receiver chamber at different timepoints for various flow rates: (i) in blue 20 μ L/min; (ii) in red 50 μ L/min; (iii) in yellow 100 μ L/min. (N = 3 independent experiments; mean ± SD).

The diffusion rate was tested under three flow rate conditions: 20 μ L/min, 50 μ L/min, and 100 μ L/min. The optimal flow rate of 50 μ L/min was identified considering greater reproducibility compared to tests conducted at a flow rate of 100 μ L/min, which were more significantly influenced by the higher pressure applied within the channels and on the membrane, as shown in Figure 3. Furthermore, this experimental conditions allowed us to reach a glucose concentration of 1 mM in the reservoir within 20 min, corresponding to a change of 18 mg/dL in blood glucose levels.

The computational model has allowed for the estimation of the diffusivity coefficient of glucose through the PC membrane, which is $6.0\times 10^{-6}\;{\text{m}}^{2}/\text{s}$ . The glucose diffusion obtained from the model within the reservoir has been evaluated at the 20-min time point, as reported in Table 1.

**TABLE 1:** Comparative analysis of experimental measurements and computational results at 20 min.

Flow rate [ μ L/min]	Simulated diffusion [mM]	Experimental diffusion [mM]
20	0.43	0.50 ± 0.18
50	0.81	0.96 ± 0.05
100	1.44	1.75 ± 0.28

**FIGURE 4:**
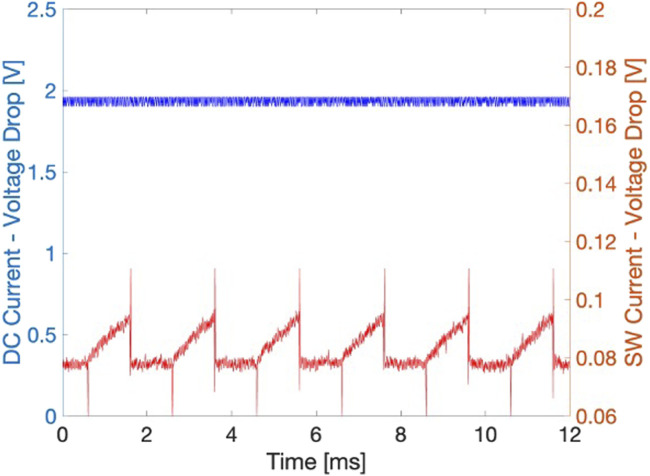
The voltage drop across the two electrodes during current-controlled electrical stimulation: in blue, the voltage at the electrodes during the application of direct current; in red, the voltage across the two electrodes during the application of the square wave current at 500 Hz, 50% duty cycle, and 5 μ A amplitude.

Subsequently, the diffusion test was repeated under current stimulation. Two stimulation patterns were applied: (i) square wave current (Figure 4 in red) and (ii) DC current (Figure 4 in blue) for 5 min. The corresponding voltage signals are shown in Figure 4. The application of an electric field enhances permeability and attracts ions in the solution toward the electrodes, which in turn facilitates the movement of neutral species, such as glucose, at physiological pH. The use of controlled square wave currents allows for lower voltage values between the electrodes due to the reduced impedance encountered at 500 Hz, as well as minimizing the Joule heating effect.

**FIGURE 5:**
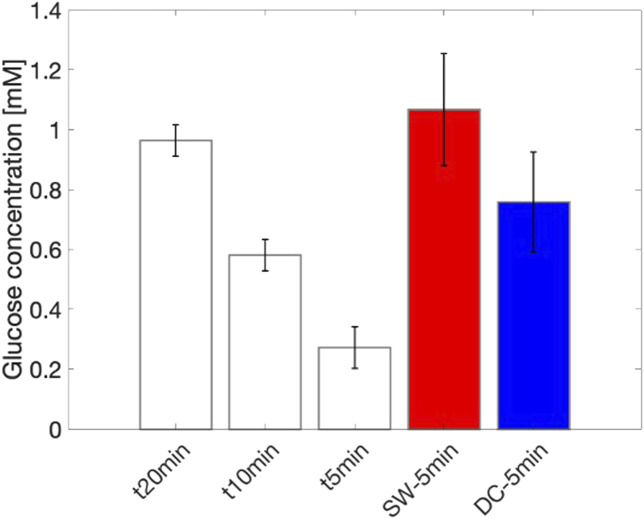
Electro-osmotic flow enhancement in diffusion dynamics: comparative analysis of diffusion mechanisms under varying applied current to enhance electro-osmotic flow. (i) In white, passive diffusion at 50 μ L/min at 5, 10, and 20 min (baseline reference); (ii) In red, diffusion with enhanced electro-osmotic flow using square wave current after 5 min; (iii) In blue, diffusion with enhanced electro-osmotic flow using DC current after 5 min.

Figure 5 shows the measured glucose concentration after 5 min under current stimulation. The diffusion achieved through DC stimulation and square wave stimulation increased of 2.8 and 3.9 times in average, respectively, compared to diffusion without stimulation. This corresponds to a passive concentration at 5 min of 0.27 ± 0.07 mM, which increases to 0.75 ± 0.16 mM and 1.06 ± 0.18 mM after stimulation for DC and square wave currents, respectively. Passive diffusion required about 20 min to reach similar concentration as the electrically assisted diffusion after 5 min, which corresponds to a reduction of the diffusion lag time by 75%. Additionally, using square wave stimulation significantly reduces the polarization load, resulting in less than 100 mV stimulation voltage compared to 2 V in DC stimulation.

**FIGURE 6:**
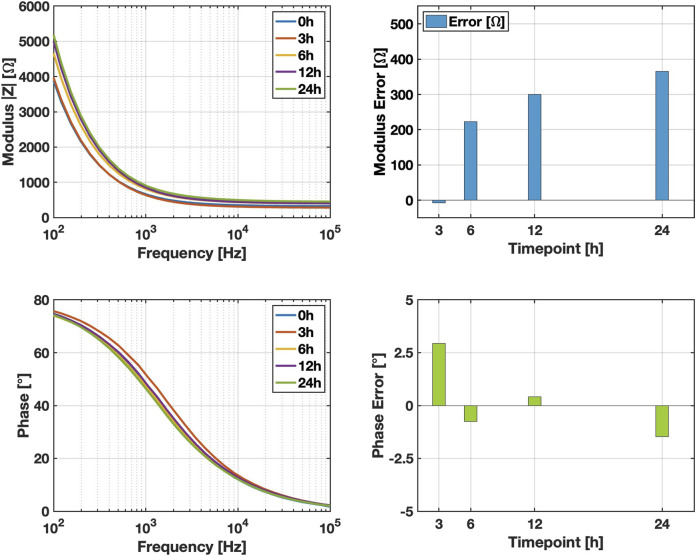
Impedance stability of electrodes under continuous stimulation. Measurements of modulus and phase were performed at 0, 3, 6, 12, and 24 h of continuous monophasic stimulation at 5 μ A. The reported errors represent the differences (Δ) relative to the values of modulus and phase at the 0 h timepoint.

Based on the obtained results, it appears that pulsed current produces effects on glucose diffusion comparable to those of DC current, consistent with findings reported in literature about Na+ transport (Bagn et al., 1990). At lower frequencies (100 Hz), medium impedance is higher, leading to larger voltage drops and increased electrode polarization, which reduces net transport. At higher frequencies ( >1 kHz), although the impedance of the medium decreases, capacitive effects dominate, causing a larger fraction of the current to be transferred along the electrode-solution interface rather than through the bulk. This reduces the effective electric field driving electro-osmotic flow and consequently lowers molecular transport efficiency. Frequencies in the intermediate range, such as the 500 Hz used in this study, therefore provide an optimal balance, maximizing electro-osmotic transport while minimizing electrode polarization and capacitive losses. To evaluate the stability of the electrodes under prolonged stimulation, we performed degradation tests at multiple time points (0, 3, 6, 12, and 24 h) under continuous monophasic stimulation at 5 μ A. The tests were designed as a stress assessment to monitor any changes in electrode impedance over time. As shown in Figure 6, the results indicate a minor increase in impedance, remaining below 400 Ω even after 24 h of continuous stimulation. These findings highlight the limited impact of sustained low-intensity current on electrode performance, confirming the robustness of the system for extended operation. Furthermore, analysis of the phase module and associated errors across time points showed minimal variation, supporting the electrodes’ stability during prolonged use.

These preliminary results lead to considerations about the possibility of leveraging electric stimulation to accelerate physiological diffusion in situations with sudden rate changes. These changes are triggered by insulin-related phenomena, stress and physical activity, where sensor response becomes critically important for patients, especially for making compensatory clinical decisions. The delay phenomena, caused by physiological diffusion lag and technological delay, can be minimized to reduce the time gap between capillary concentration and ISF concentration. Moreover, the technological delay persists in the measurement delay contribution due both to the response times of the enzymatic sensor and to the computational times. This delay contribution can be reduced by introducing a non-enzymatic glucose sensor to construct a fully robust system (Wei et al., 2020; Hwang et al., 2018; Abbasnia et al., 2024; Franceschini and Taurino, 2022; Franceschini et al., 2023).

Although the proposed microfluidic platform provides a controlled and reproducible environment to investigate magnitude gradient of glucose transport, it represents a simplified model of the complex architecture of the dermal interstitial space diffusion. In particular, static reservoirs and the polycarbonate membrane do not fully reproduce the heterogeneous extracellular matrix present *in vivo*, which includes collagen networks, binding proteins, and cellular components that influence diffusion and fluid dynamics. These structural and biochemical features are not captured in the current model, constituting an intrinsic limitation.

However, it is important to note that the primary objective of this study was not to fully replicate the *in vivo* dermal environment, but rather to validate the concept that low-intensity electrical stimulation can enhance glucose transport across a defined barrier. These simplified system allows precise control over experimental variables and the isolation of electro-osmotic effects without confounding factors from cellular metabolism or matrix complexity.

Moreover, the diffusion delays measured in our system under passive conditions are within the range reported in continuous glucose monitoring studies (15–20 min), supporting the translational relevance of the model. For future work, more physiologically representative platforms could include collagen-based hydrogels with cellular components, which would better mimic the mechanical, tortuous, and reactive nature of the interstitial space. Despite these simplifications, the present *in vitro* model effectively demonstrates the potential of pulsed electrical currents to accelerate glucose transport, providing a foundation for future bioengineered systems.

## 4 Conclusion

In the present work, we have presented and validated a novel approach to reduce the delay present in commercial CGMs caused by physiological diffusion lag time between capillary blood and ISF. To achieve this, we fabricated an experimental setup, which allowed us to obtain experimental results of glucose diffusion rates under current stimulation. We demonstrated that using a low current density of 140 μ A/cm^2^, which is almost 4 times lower than the upper safety guideline limit, enabled us to reduce glucose diffusion time between compartments simulating capillary blood and ISF by 75%, from 20 min to 5 min. Furthermore, for the same current, the importance of square wave stimulation was highlighted, showing comparable performance to DC stimulation. The benefits of square wave current stimulation included reduced Joule heating and, above all, a lower applied potential between the two electrodes.

Although our microfluidic setup offers important insights into glucose diffusion under electrical stimulation, it represents an approximation of the *in vivo* dermal environment. Future studies will focus on validating these findings in more physiologically relevant models.

The proposed technology can be optimized to simulate a continuous trend throughout the day. In this way, we can evaluate the effects of stimulation techniques on intradermal diffusion, allowing for significant progress toward lag-free glucose sensors. Beyond diabetes applications, this technology can be integrated into any minimally invasive sensor to reduce the lag time and improve sensor accuracy in monitoring molecules diffusing from capillary blood to ISF.

Beyond diabetes applications, this proof-of-concept can be integrated into any minimally invasive sensor to reduce lag time and improve accuracy in monitoring molecules diffusing from capillary blood to ISF. For practical wearable applications, future developments will focus on miniaturization and integration with CGM sensors. In addition, the impact of electrical stimulation on sensor performance will be assessed to ensure safe operation and reliable biosensing.

## Data Availability

The raw data supporting the conclusions of this article will be made available by the authors upon request.
